# Is Early Oral Feeding after Gastric Cancer Surgery Feasible? A Systematic Review and Meta-Analysis of Randomized Controlled Trials

**DOI:** 10.1371/journal.pone.0112062

**Published:** 2014-11-14

**Authors:** Xiaoping Liu, Da Wang, Liansheng Zheng, Tingyu Mou, Hao Liu, Guoxin Li

**Affiliations:** 1 Department of General Surgery, Nanfang Hospital, Southern Medical University, Guangzhou, Guangdong, P.R. China; 2 Department of Gastrointestinal Surgery, The first affiliated hospital of Gannan medical university, Gannan medical university, Ganzhou, Jiangxi, P.R. China; University Hospital Oldenburg, Germany

## Abstract

**Aim:**

To assess the feasibility and safety of early oral feeding (EOF) after gastrectomy for gastric cancer through a systematic review and meta-analysis based on randomized controlled trials.

**Methods:**

A literature search in PubMed, Embase, Web of Science and Cochrane library databases was performed for eligible studies published between January 1995 and March 2014. Systematic review was carried out to identify randomized controlled trials comparing EOF and traditional postoperative oral feeding after gastric cancer surgery. Meta-analyses were performed by either a fixed effects model or a random effects model according to the heterogeneity using RevMan 5.2 software.

**Results:**

Six studies remained for final analysis. Included studies were published between 2005 and 2013 reporting on a total of 454 patients. No significant differences were observed for postoperative complication (RR = 0.95; 95%CI, 0.70 to 1.29; *P* = 0.75), the tolerability of oral feeding (RR = 0.98; 95%CI, 0.91 to 1.06; *P* = 0.61), readmission rate (RR = 1; 95%CI, 0.30 to 3.31; *P* = 1.00) and incidence of anastomotic leakage (RR = 0.31; 95%CI, 0.01 to 7.30; *P* = 0.47) between two groups. EOF after gastrectomy for gastric cancer was associated with significant shorter duration of the hospital stay (WMD = −2.36; 95%CI, −3.37 to −1.34; *P*<0.0001) and time to first flatus (WMD = −19.94; 95%CI, −32.03 to −7.84; *P* = 0.001). There were no significant differences in postoperative complication, tolerability of oral feeding, readmission rates, duration of hospital stay and time to first flatus among subgroups stratified by the time to start EOF or by partial and total gastrectomy or by laparoscopic and open surgery.

**Conclusions:**

The result of this meta-analysis showed that EOF after gastric cancer surgery seems feasible and safe, even started at the day of surgery irrespective of the extent of the gastric resection and the type of surgery. However, more prospective, well-designed multicenter RCTs with more clinical outcomes are needed for further validation.

## Introduction

Recently, the concept of fast-track surgery is drawing increasing attention, which requires multidisciplinary team work to accelerate recovery during perioperative care [1]. Early oral feeding (EOF) is one of the most important parts of fast-track surgery elements. The advantages of early enteral nutrition after colorectal surgery have been demonstrated in several reports, such as a shorter length of hospital stay and less postoperative morbidity and mortality compared with traditional postoperative oral feeding (TOF) [2]–[4].

Gastric cancer is the second most common cause of cancer-related death worldwide, of which the global incidence is declining. However, there remains quite higher morbidity rate in Asia compared to that in western countries [5]. Gastric cancer can be cured successfully with surgical resection and the operative technique and anastomotic type for gastric cancer has been gradually standardized [6]–[8]. Then, surgeons should pay more attention to how to enhance recovery, reduce complications and improve quality of life of patients undergoing gastrectomy for gastric cancer [9]–[11]. To date, the introduction of fast-track surgery following gastrectomy has been demonstrated for nearly one decade [12]–[14]. However, as a key element of fast track surgery pathway, the significance of EOF after gastric cancer surgery is still controversial.

The purpose of this study was to assess the feasibility and safety of EOF in patients after gastric cancer surgery through a systematic review based on randomized controlled trials.

## Materials and Methods

### Search strategy

The relevant literature was searched from PubMed, Embase, Web of Science and Cochrane library databases published between January 1995 and March 2014. The following search terms was used: gastric cancer, gastrectomy, early oral feeding, early oral intake, enhanced recovery and fast-track surgery. Reference lists within selected studies and abstracts published at major international conferences were also searched for potentially eligible studies.

### Study inclusion criteria

The studies were limited to be described as the design type of randomized controlled trials (RCTs) with or without blinding method, comparing EOF with TOF following gastrectomy for gastric cancers. Oral feeding was following a stepwise plan from water to other liquids to semi-fluids to normal food. EOF was defined as oral feeding of water or glucose saline initiated before flatus as tolerated; TOF was defined as oral feeding initiated certainly after flatus. Comparative studies that included patients undergoing EOF after gastric cancer surgery through nasogastric enteral nutrition were excluded. We applied restrictions with respect to language in English.

### Quality Assessment

The quality of included RCTs was assessed by two reviewers (XP Liu and D Wang) independently according to the Cochrane Collaboration's tool for assessing risk of bias, which addressed seven items: random sequence generation, allocation concealment, blinding of participants and personnel, blinding of outcome assessment, incomplete outcome data, freedom from selective reporting, and freedom from other bias [15]. Disagreement was resolved by consensus and discussion.

### Outcomes

The primary outcomes were postoperative complication, tolerability of oral feeding and readmission rates. The secondary outcomes were the duration of hospital stay expressed as hospitalization days after surgery, time of first flatus and incidence of anastomotic leakage. Patients failed to tolerate EOF presented as recurrent nausea, vomiting, abdominal distension without intestinal sound or nasogastric tube was reinserted.

### Statistical analysis

Statistical analysis was performed with Cochrane Collaboration's RevMan5.2 software (Cochrane Collaboration, Oxford, UK). For continuous data, results from each study were expressed as a weighted mean difference (WMD) with 95% confidence intervals (CI) and combined for meta-analysis. Data were summarized graphically in forest plots. For dichotomous data, results for each study were performed by using the relative risk (RR). The Mantel–Haenszel method was used to combine the RRs for the outcomes of interest. A funnel plot for postoperative complication was constructed to evaluate publication and other biases [16].

Heterogeneity was measured through *X* ^2^ and I^2^ test. If between-study heterogeneity existed (I^2^>50%), random-effect model was used; otherwise, meta-analysis was done with fixed effect model. *P*<0.05 in two-sided test was considered statistically significant.

## Results

### Selected studies

**Figure 1:**
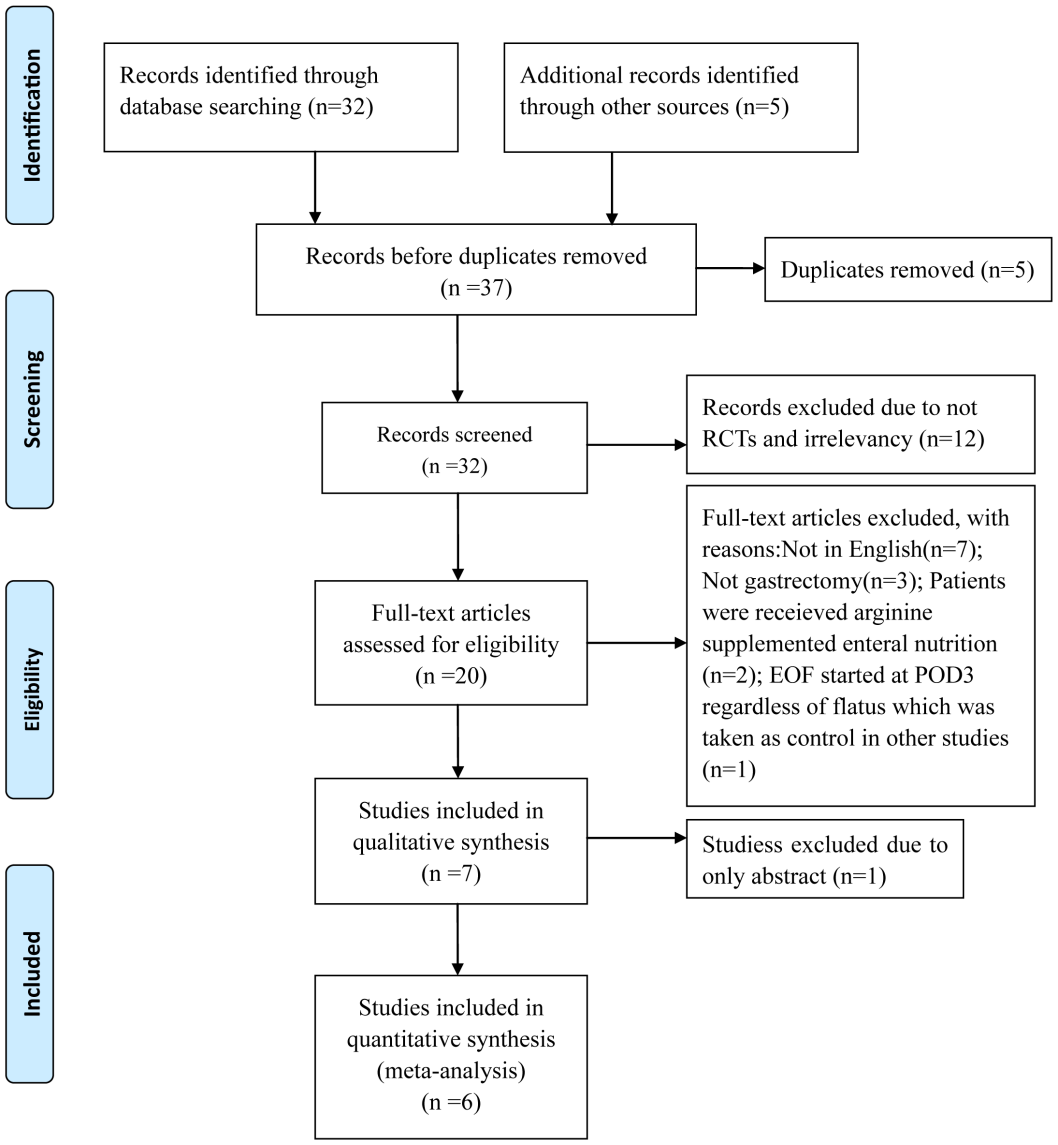
Flow chart of studies identified, included and excluded following PRISMA statement.

A flow chart detailing the process of study identification and selection following the PRISMA statement (see Checklist S1) is shown in Fig.1. Six studies finally remained for further analysis. Included studies were published between 2005 and 2013 and reported on a total of 454 patients [17]–[22]. In the study by Chen *et al.*, they carried out subgrourp analysis stratified by laparoscopic and open surgery, so L and O were used to tell them apart in the present meta-analysis [17]. The characteristics of these six studies are summarized in Table 1.

**Table 1:** Characteristics of the studies included in meta-analysis.

Author	Country	Year	Time of oral feeding	Extent of gastrectomy	Group	Number	Age	Gender	BMI	ASA	TNM stage
							(years)	(m/f)	(kg/m^2^)	(0/1/2)	(I+II)/(III+IV)
Liu[21]	China	2010	day of surgery	TG, PG, DG	EOF	30	60.7±9.7	18/15	21.84±2.65	2^c^	8/25
					TOF	33	61.9±8.3	15/14	21.28±2.54	2 c	7/23
Wang[22]	China	2010	day of surgery	PG, DG	EOF	45	58.76±9.66	32/13	23.85±2.40	NR	NR
					TOF	47	56.87±9.16	29/18	23.25±2.79		
Hur[19]	Korea	2011	POD1	TG, SG	EOF	28	24/4 a	20/8	21/7 b	10/18^d^	NR
					TOF	26	21/5^a^	13/13	19/7^b^	4/22^d^	
Chen(L)[17]	China	2012	6–8*h* after surgery	DG	EOF	19	59(49–71)	10/9	22.94±2.23	NR	11/8
					TOF	22	62.5(45–72)	10/12	22.99±2.24		11/11
Chen(O)[17]	China	2012	6–8*h* after surgery	DG	EOF	21	64(40–71)	9/12	23.54±2.59	NR	9/12
					TOF	20	64.5(49–75)	12/8	23.47±2.62		7/13
Kim[20]	Korea	2012	POD2	DG	EOF	22	52.64±11.57	13/9	23.40±3.17	0/14/8	21/1
					TOF	22	57.45±14.54	15/7	23.77±3.54	0/14/8	22/0
Feng[18]	China	2013	day of surgery	TG	EOF	59	54.98±11.35	41/18	22.44±3.51	0/3/56	26/33
					TOF	60	55.79±10.06	44/16	21.01±1.78	0/1/59	39/21

BMI: body mass index; ASA: American Society of Anesthesiology; TNM: tumor, node, metastasis; NR: not reported; POD: postoperative day; TG: total gastrectomy; DG: distal gastrectomy; PG: proximal gastrectomy; SG: subtotal gastrectomy;

a : Age grouped by <65/≥65;

b : BMI grouped by <25 kg/m^2^/≥25 kg/m^2^;

c : median;

d : ASA score grouped by 0/(1 and 2).

### Methodological quality of the studies

**Figure 2:**
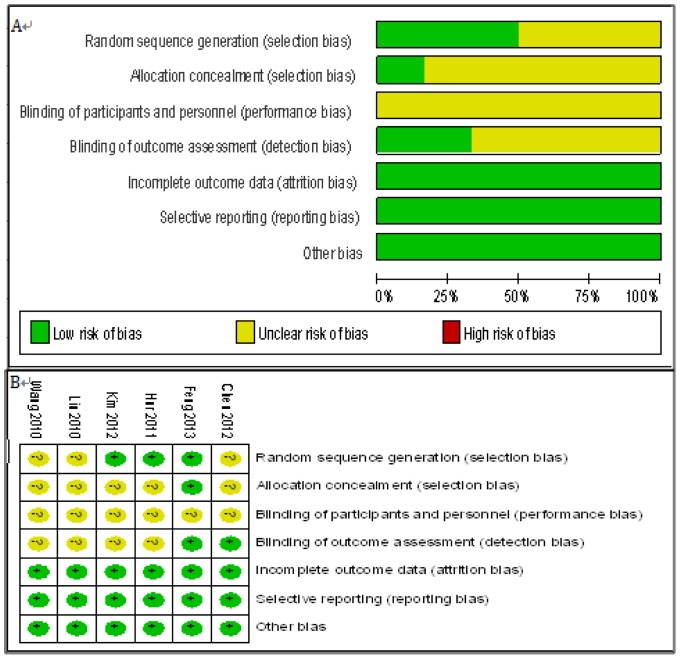
Risk of bias graph. Judgements about each risk of bias item presented in all include RCTs(A) and for each included RCT(B).

Risk of bias was assessed by the Cochrane Collaboration's tool. As concerns for early oral feeding being compared, blinding was inherent defect for such studies. Generally, the included studies had a moderate risk of bias (Fig.2).

### Primary outcome parameters

#### Postoperative complication

**Figure 3:**
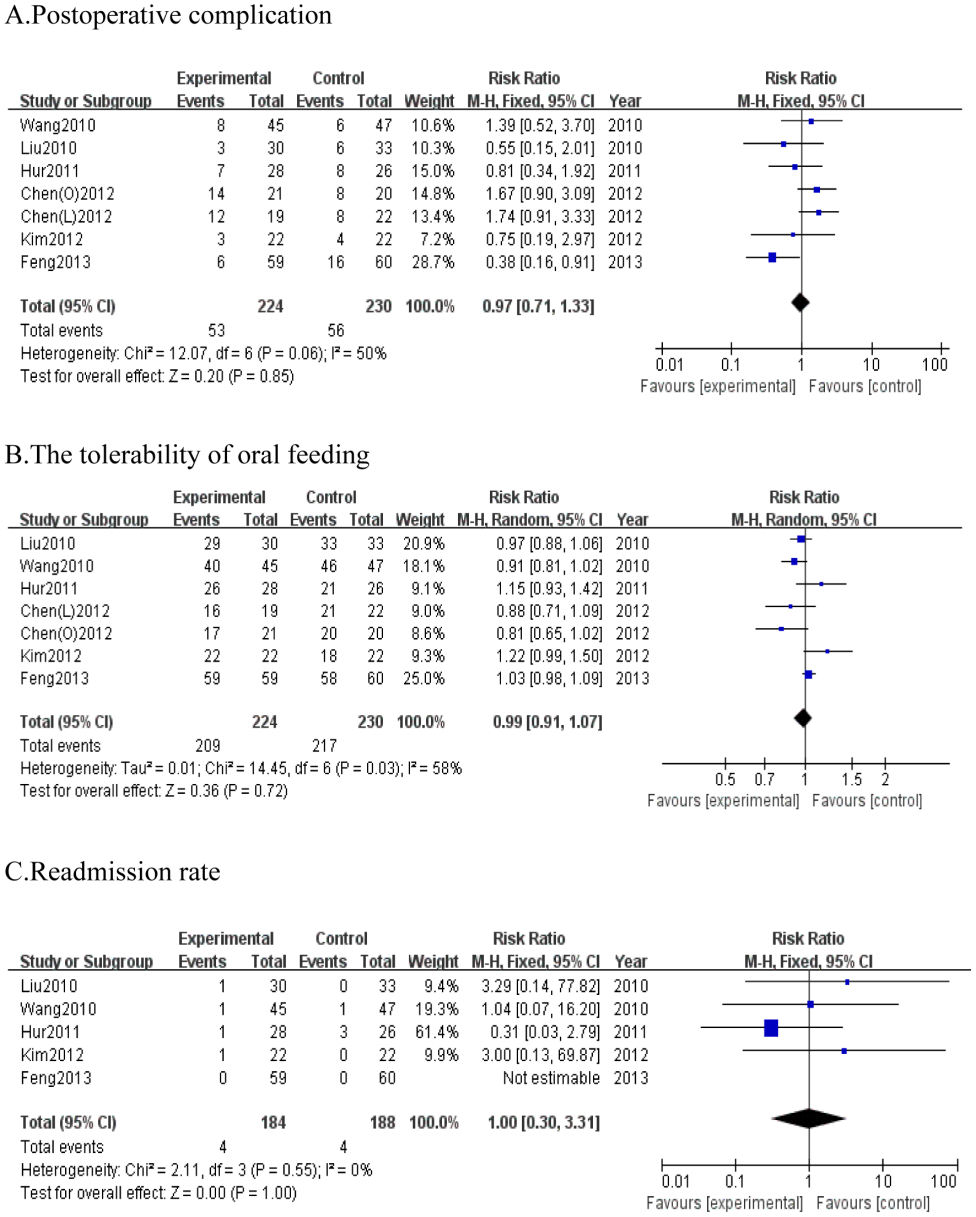
Forest plot displaying the results of the meta-analysis. A. postoperative complication; B. the tolerability of oral feeding; C. readmission rate. RR: Risk ratio; WMD: Weighted mean difference. CI: confidence intervals.

Primary outcome for this systematic review was postoperative complication. All six studies provided information on postoperative complication. The pooled results indicated no evidence of a significant difference in the number of complications between two groups (RR = 0.97; 95% CI, 0.71 to 1.33; *P* = 0.85), and there was no remarkable heterogeneity among studies (*P* = 0.06, I^2^ = 50%) (Fig.3A).

#### The tolerability of oral feeding

Six studies were all included with 224 and 230 cases in each group respectively. Among them, 209 patients tolerated EOF while 15 failed. Using a random effects model, the pooled results showed that there was no significant difference between two groups about tolerability of oral feeding after gastrectomy (RR = 0.99; 95%CI, 0.91 to 1.07; *P* = 0.72), with obvious heterogeneity (*P* = 0.03, I^2^ = 58%) (Fig.3B).

#### Readmission rate

In the five studies [18]–[22] reporting on readmission rate after gastric cancer surgery with 372 patients, the pooled readmission rate was similar between both groups based on fix effects model analysis (RR = 1; 95%CI, 0.30 to 3.31; P = 1.00), without significant heterogeneity (*P* = 0.55, I^2^ = 0%) (Fig.3C).

### Secondary outcome parameters

#### The duration of hospital stay

**Figure 4:**
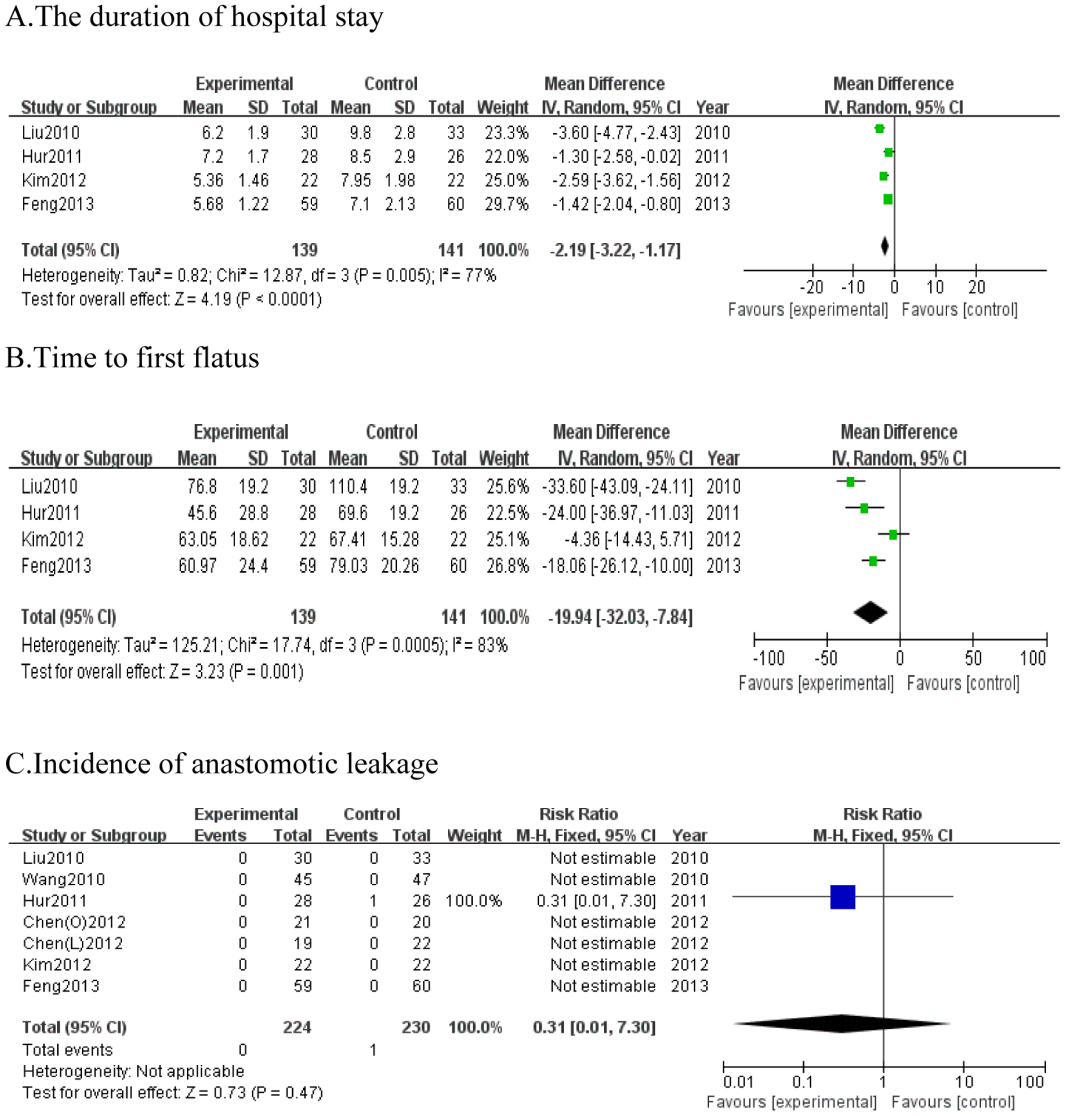
Forest plot displaying the results of the meta-analysis. A. the duration of hospital stay; B. time of first flatus; C. incidence of anastomotic leakage. RR: Risk ratio; WMD: Weighted mean difference. CI: confidence intervals.

All included studies reported information on the duration of hospital stay, however, two studies, Chen *et al.* [17] and Wang *et al.* [22] only described the median postoperative hospital stay. So, we excluded them out of this meta-analysis. The duration of hospital stay was significantly shorter with EOF than TOF in all included studies (WMD = −2.19; 95%CI, −3.22 to −1.17; *P*<0.001). Pooled analysis of the duration of hospital stay showed obvious heterogeneity between studies (*P* = 0.005, I^2^ = 77%) (Fig.4A).

#### Time to first flatus

Four studies [18]–[21] provided the complete information on time to first flatus. Time to first flatus was significantly shorter for EOF group than TOF group after gastric cancer surgery (WMD = −19.94; 95% CI, −32.03 to −7.84; *P* = 0.001). Because of significant heterogeneity (*P* = 0.0005, I^2^ = 83%), a random effects model was used (Fig.4B).

#### Incidence of anastomotic leakage

Anastomotic leakage is one of the major postoperative complications. We observed that the incidence of anastomotic leakage was comparable for two groups among all patients as similar in all included studies (RR = 0.31; 95%CI, 0.01 to 7.30; *P* = 0.47) (Fig.4C). There was only one patient suffered anastomotic leakage in Hur *et al.*'s study [19]. So the heterogeneity was not applicable.

#### Sensitivity analysis

We reanalyzed the primary outcome parameters by including only studies with total sample size no smaller than 50 in order to perform sensitivity analysis. The results were not substantially influenced by sensitivity analysis as shown in Table 2. The results of sensitivity analysis supported the credibility of the evidence in this meta-analysis.

**Table 2:** Sensitivity analysis results of primary outcomes by included studies of no less than 50 patients in each group.

Primary outcomes	Number of studies	Patients	WMD/RR	95%CI	Analysis model	*P*	heterogeneity	
		(EOF/TOF)					I^2^	*P*
postoperative complication	5	202/208	0.90	0.47, 1.70	Random	0.73	66%	0.02
Tolerability of oral feeding	5	202/208	0.97	0.89, 1.05	Random	0.47	67%	0.02
Readmission rates	4	162/166	0.78	0.20, 3.00	Fixed	0.72	0%	0.47

RR: Risk ratio; WMD: Weighted mean difference. CI: confidence intervals.

#### Subgroup analysis

Subgroup analysis according to the time of starting EOF, extent of gastrectomy and type of surgery, were also performed to assess potential effect modification of these variables on outcomes. Tables 3–5 show the results of subgroup analysis. The similar outcomes could be observed in the stratified groups irrespective of the time to start EOF, the extent of gastric resection and the type of surgery.

**Table 3:** Results of subgroup analysis comparing the time of EOF after gastrectomy.

Outcomes	Subgroup	Number of studies	Patients	WMD/RR	95%CI	*P*	Heterogeneity	
			(EOF/TOF)				I^2^	*P*
Postoperative complication	Day of surgery	5	174/182	1.02	0.72, 1.45	0.91	64%	0.02
	Day after surgery	2	50/48	0.79	0.38, 1.65	0.53	0%	0.92
Tolerability of oral feeding	Day of surgery	5	174/182	0.94	0.86, 1.03	0.21	67%	0.02
	Day after surgery	2	50/48	1.18	1.02, 1.37	0.03	0%	0.71
Readmission rates	Day of surgery	3	134/140	1.78	0.24, 13.09	0.57	0%	0.59
	Day after surgery	2	50/48	0.68	0.14, 3.28	0.63	26%	0.25
Duration of hospital stay	Day of surgery	2	89/93	−2.45	−4.58,−0.32	0.02	90%	0.001
	Day after surgery	2	50/48	−2.00	−3.26,−0.74	0.002	58%	0.12
Time to first flatus	Day of surgery	2	89/93	−25.62	−40.84,−10.40	0.001	83%	0.01
	Day after surgery	2	50/48	−13.74	−32.96, 5.49	0.16	82%	0.02

RR: Risk ratio; WMD: Weighted mean difference. CI: confidence intervals.

Day of surgery: 6 h≤ time to start EOF≤ 24 h after gastrectomy;

Day after surgery: time to start EOF>24 h after gastrectomy.

**Table 4:** Results of subgroup analysis comparing EOF in TG and SG for gastric cancer.

Outcomes	Subgroup	Number of studies	Patients	WMD/RR	95%CI	*P*	Heterogeneity	
			(EOF/TOF)				I^2^	*P*
Postoperative complication	TG	1	59/60	0.38	0.16, 0.91	0.03	Not applicable	
	SG	4	107/111	1.54	1.04, 2.28	0.03	0%	0.72
Tolerability of oral feeding	TG	1	59/60	1.03	0.98, 1.09	0.25	Not applicable	
	SG	4	107/111	0.94	0.81, 1.09	0.44	63%	0.05
Duration of hospital stay	TG	1	59/60	−1.42	−2.04,−0.80	<0.00001	Not applicable	
	SG	1	22/22	−2.59	−3.62,−1.56	<0.00001	Not applicable	
Time of first flatus	TG	11	59/60	−18.06	−26.12,−10.00	<0.0001	Not applicable	
	SG	1	22/22	−4.36	−14.43, 5.71	0.40	Not applicable	

TG: total gastrectomy; SG: subtotal gastrectomy; RR: Risk ratio; WMD: Weighted mean difference. CI: confidence intervals.

**Table 5:** Results of subgroup analysis comparing EOF in Laparoscopy and open surgery for gastric cancer.

Outcomes	Subgroup	Number of studies	Patients	WMD/RR	95%CI	*P*	Heterogeneity	
			(EOF/TOF)				I^2^	*P*
Postoperative complication	Laparoscopy	2	41/44	1.39	0.77, 2.51	0.27	18%	0.27
	Open	5	183/186	0.86	0.59, 1.25	0.43	57%	0.06
Tolerability of oral feeding	Laparoscopy	2	41/44	1.04	0.76, 1.42	0.82	77%	77%
	Open	5	183/186	0.97	0.90, 1.06	0.54	61%	0.04
Readmission rates	Laparoscopy	1	22/22	3.00	0.13, 69.87	0.49	Not applicable	
	Open	4	162/166	0.78	0.20, 3.00	0.72	0%	0.47
Duration of hospital stay	Laparoscopy	1	22/22	−2.59	−3.62,−1.56	<0.001	Not applicable	
	Open	3	117/119	−2.07	−3.45, −0.70	0.003	82%	0.004
Time of first flatus	Laparoscopy	1	22/22	−4.36	−14.43, 5.71	0.40	Not applicable	
	Open	3	117/119	−25.07	−35.05, −15.09	<0.001	67%	0.05

RR: Risk ratio; WMD: Weighted mean difference. CI: confidence intervals.

#### Publication bias

**Figure 5:**
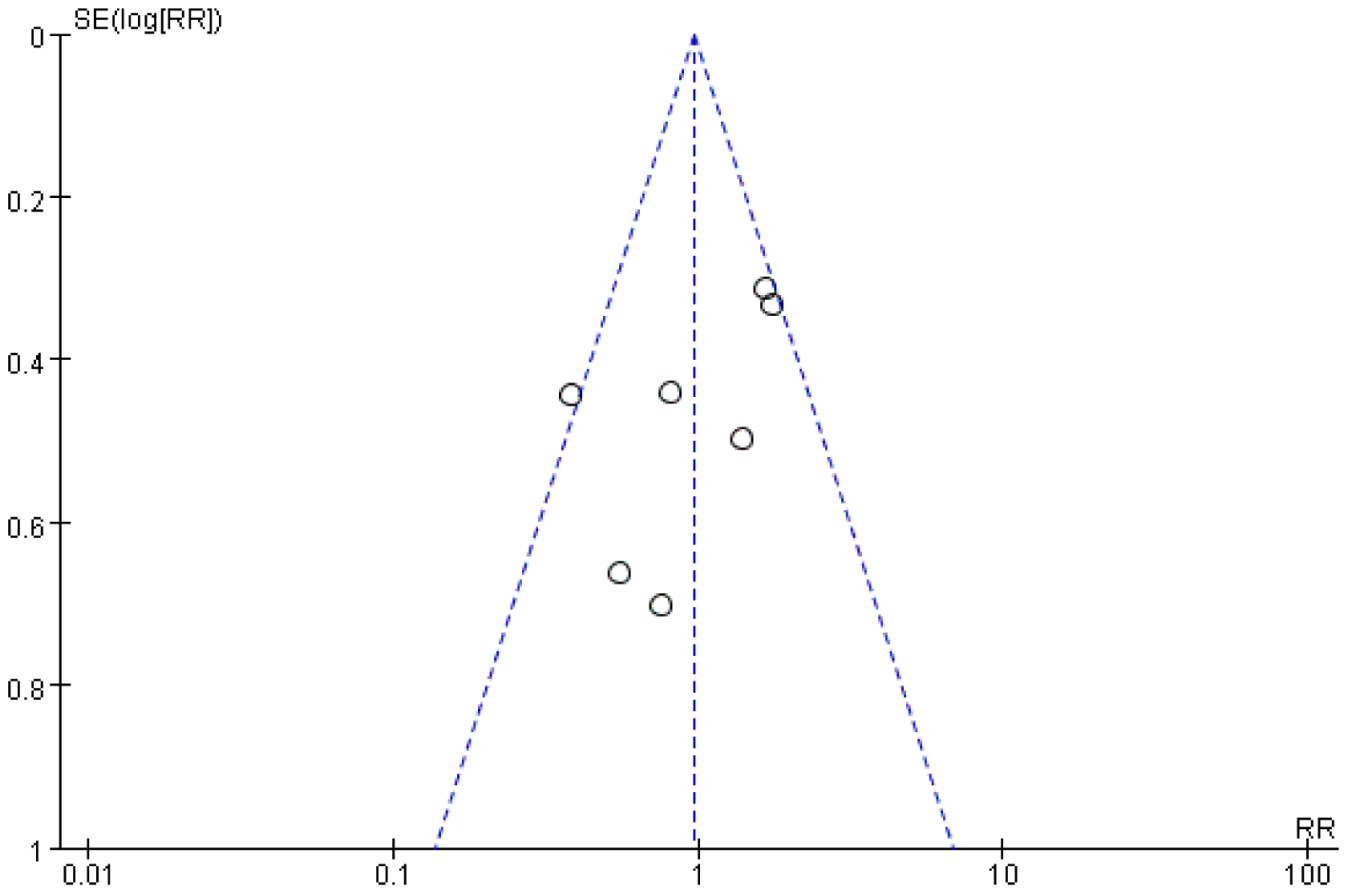
Funnel plot of the studies reporting on postoperative complication. RR: risk ratio; SE: standard error.

Publication bias was evaluated by performing a funnel plot of postoperative complication (Fig.5). All included studies reported on this outcome, which were equally distributed on the vertical axis, the result showed no evidence of obvious publication bias.

## Discussion

The present meta-analysis showed that early oral feeding (EOF) did not increase postoperative complication, readmission rate and the incidence of anastomotic leakage, either. We found no significant difference in tolerability of oral feeding after gastrectomy between both groups. Furthermore, EOF following gastric surgery was associated with a significant reduction in duration of postoperative hospital stay and time to first flatus compared with TOF.

Though emerging evidence for the advantages of EOF has been demonstrated, many surgeons are still reluctant to administer EOF to patients after gastrectomy. One reason is fearing an increase of postoperative complications, such as gastric retention and anastomosis dehiscence[23], [24]. Regarding the safety of EOF, previous evidence from a systematic review in patients undergoing colonic surgery has shown that EOF is safe without any significant increase in complications [25], [26]. What's more, in a meta-analysis of studies comparing early enteral feeding versus “nil by mouth”, those patients receiving enteral nutrition had a lower incidence of infection complications undergoing gastrointestinal surgery [27]. The major factor used to justify the traditional practice of oral intake restriction and EOF after gastrectomy is concern for anastomosis leakage [28]. However, restricting EOF is not evidence-based. On the contrary, EOF after upper gastrointestinal surgery was found to promote anastomotic healing and anastomotic strength in intestines and somatic tissues of a rat model [29]. Early postoperative oral diet is easily absorbed, which may accelerate recovery of peristalsis, protect gut mucosal barrier function, and strengthen immune response [30]. Traditional nutritional routes still adhere to the first flatus with postoperative fasting, decompression of nasogastric tube and the supply of a large number of intravenous fluids for several days after gastrectomy. Thus, some pulmonary complications such as atelectasis, pneumonia, gastroesophageal reflux in patients may be induced [31]. In the present meta-analysis, we observed no significant differences between two groups concerning postoperative complications, including the presence of anastomotic leakage.

Inducing gastrointestinal symptoms is another concern to justify EOF restriction after gastrectomy [32], [33]. It was supposed that EOF might result in the risk of increasing postoperative nausea and vomiting [4], [34], [35]. Thus, the patients would not only suffer from intolerance of EOF, but also encounter severe adverse events. Traditionally, the time to resume diet depends on the passage of flatus. However, such an approach was considered extremely conservative through the physiology research of postoperative ileus [36]. Difronzo *et al.* [37] showed that over 80% of patients tolerated EOF after colonic surgery by analyzed 200 patients during a five-year period. From all included studies of this meta-analysis, in spite that nausea, vomitting or abdominal distension occurred in some patients receiving EOF, the symptoms mostly happened in the initial stage of oral diet and did not develop into severe complications. The pooled data showed that there was no significant difference between two groups about tolerability of oral feeding after gastrectomy, and EOF didn't increase the risk of postoperative morbidity and mortality. Therefore, removing a nasogastric tube and early oral intake after gastrectomy as soon as possible is a considerable strategy for postoperative patients [38], [39]. Moreover, on the basis of safety and tolerability of EOF, we also see from this meta-analysis, EOF might further shorten the hospital stays, lessen first flatus time and didn't increase the patient's readmission to the hospital. It was considered that EOF following gastrectomy could benefit patients.

From the studies included in this meta-analysis, various time of EOF after surgery between studies was found such an 6 to 8 hours after surgery, the first postoperative day and the third postoperative day et al. Jeong *et al.* [40] reported EOF was safe and feasible on the first postoperative day after gastrectomy, however, an old age (≥70 years) required careful monitoring when applying EOF after surgery. Lewis *et al.* [41] compared early enteral nutrition within 24 *h* of gastrointestinal surgery versus later commencement of feeding, it was found that mortality was reduced with early postoperative feeding even though increased vomiting. Thus, the given time of EOF after surgery is still controversial. In our included studies, time of EOF after gastrectomy was mostly based on an accelerated rehabilitation protocol designed for colorectal resection surgery. According to the time of EOF, we divided the included studies into two subgroup, day of surgery subgroup [17], [18], [21], [22] and day after surgery subgroup [19], [20]. Most outcomes in EOF were found similar with TOF in the stratified subgroups consistent with the pooled analysis, which somewhat suggested that to start EOF at 6 or 8 hours after surgery might be safe, in spite of small sample studies contributing to it.

Simultaneously, we also analyzed the outcomes stratified into total gastrectomy (TG) subgroup [18] and subtotal gastrectomy (SG) subgroup [17], [20], [22]. Similar findings were found in both TG and SG group with regard to tolerability of oral feeding, duration of hospital stay and time of first flatus except postoperative complication. For TG, the incidence of postoperative complications seemed lower in EOF group than that in TOF group, which turned out to be opposite for SG. The possible reasons underlying the distinctions between TG and SG might be that the extent of the gastric resection decided types of digestive tract reconstruction, which led to different effects on postoperative physiological functions, like gut motility and metabolism. Thus, some minor gastrointestinal symptoms such as abdominal cramps, colic, nausea and vomiting would be induced, especially in SG which preserved the function of gastric acid secretion, resulting in increased overall postoperative complications. However, no major complications like anastomotic leakage were observed in most studies that had reported on this issue. Besides, it was too difficult to reach a conclusive outcome based on pooled analysis including only two studies with quite small sample sizes. On the whole, no obvious change was observed regarding the primary outcomes of the present meta-analysis. It might still be feasible under careful assessment for both TG and SG. And similar findings were also observed in the subgroup analyses stratified by laparoscopic and open surgery. It is believed that postoperative recovery of bowel motion could be affected by abdominal incision size. Patients in laparoscopy group are supposed to have a better recovery for the intrinsic advantages of minimally invasive surgery over conventional one. Unfortunately, due to limited sample size, such conclusions could not be draw out of the given data. So a large-scale well-designed RCT is warranted to clarify this difference more conclusively. However, in this meta-analysis EOF seemed acceptable for patients in both laparoscopic and open surgery group.

Several limitations were associated with included randomized studies deserving consideration in the interpretation of this meta-analysis. First, small sample size, single-center experience and moderate quality of included studies might decrease the reliability of the results. Second, insufficient background of clinical information, differences in operating technique, perioperative nursing system and outcomes examined were discovered in our included studies. Third, obvious bias in population was found. Most of the studies were done in East Asian countries. However, the studies of white and black population were lacked. As widely known, factors such as dietary history, preoperative obesity could also influence the EOF on patients after gastric cancer surgery. Thus, given the above defects, different strategies were used to eliminate bias. Then, subgroup analysis was performed to detect potential bias sources, stratifying the time to start EOF, the extent of the gastric resection and the type of surgery to acquire robust evidence for the conclusions. All these attempts supported the credibility of the evidence in this meta-analysis.

In conclusion, this meta-analysis supported that EOF after gastric cancer surgery seemed feasible and safe, even started at the day of surgery irrespective of the extent of the gastric resection and the type of surgery. However, more prospective, well-designed multicenter RCTs with more clinical outcomes are needed for further validation.

## Supporting Information

Checklist S1 **PRISMA Checklist.**
(DOC)
